# Prolonged delusional state triggered by repeated ingestion of aromatic liquid in a past 5-methoxy-*N*, *N*-diisopropyltryptamine abuser

**DOI:** 10.1186/1940-0640-8-9

**Published:** 2013-04-11

**Authors:** Yasuko Fuse-Nagase, Toru Nishikawa

**Affiliations:** 1 University Health Center, Ibaraki University, 2-1-1 Bunkyo, Mito, Ibaraki 310-8512, Japan; 2 Section of Psychiatry and Behavioral Sciences, Tokyo Medical and Dental, University Graduate School, 1-5-45 Yushima, Bunkyo-ku, Tokyo 113-8519, Japan

**Keywords:** Drug abuse, Delusional state, 5-MEO-DIPT, Liquid aromatics, Sensitization

## Abstract

A 30-year-old Japanese man with no previous psychiatric history presented to our facility with delusions, which had been ongoing for 2 months. Upon further interview, he confided that he had a past history of recurrent 5-methoxy-*N*,*N*-diisopropyltryptamine (5-MeO-DIPT or “Foxy”) abuse, as well as a recent history of recurrent ingestion of a legal aromatic liquid used as a recreational drug. After this episode, his condition improved and he did not follow up at subsequent appointments. However, 6 months later, he suffered a relapse of prolonged delusions after again ingesting a recreational aromatic liquid. An evaluation of the chronological sequence of the patient’s condition revealed that ingestion of these aromatic liquids, which can be purchased easily on the Internet, likely triggered the patient’s delusional episodes. We speculate that the patient’s recurrent abuse of 5-MeO-DIPT caused sensitization (or reverse tolerance), thus prolonging his delusions. Sensitization is the amplification of a response following repeated administrations of a stimulus. 5-MeO-DIPT is a popular drug of abuse, and it is highly probable that a large number of past 5-MeO-DIPT users are currently sensitized. This is an important latent factor underlying subsequent episode of drug-induced psychosis. Psychiatrists should consider the possibility of 5-MeO-DIPT sensitization when evaluating patients with acute psychoses.

## Background

Abuse of commercially available incense, herbs, bath salts, and aromatic liquids is a common social problem in many countries [1,2]. Here, we report a case of a patient with a prolonged delusional episode after ingesting a legal aromatic liquid. To the best of our knowledge, there are no prior reports of prolonged delusions triggered by abusing these commercially available substances. In addition, the patient had a past history of 5-methoxy-*N*,*N*-diisopropyltryptamine (5-MeO-DIPT or “Foxy”) abuse, a hallucinogenic drug widely abused and now illegal in many countries. We speculate that his past history of 5-MeO-DIPT abuse was related to the prolongation of his delusions.

## Case presentation

### Case description

A 30-year-old Japanese man with a normal developmental history, and no personal or family history of prior psychiatric disease, presented with delusions in May 2011. The patient was a university graduate and employed at a company. His past history included tuberculosis at age 27 years old. His delusions consisted of persistent paranoid beliefs that somebody was conducting audio surveillance of his house and that his wife was having an affair, and he became obsessed with trying to find hidden listening devices and tracking his wife’s whereabouts. Worried about the possibility of schizophrenia, his family brought him for evaluation. Schizophrenia was ruled out since he did not meet diagnostic criteria [3]: his psychiatric symptoms lasted less than 6 months at first visit, he had no hallucinations, his speech was not disorganized, his behavior was neither disorganized nor catatonic, and he showed no clear negative symptoms. The patient was empirically started on oral olanzapine 5 mg/day.

The patient’s psychotic symptoms improved over the following three months. During follow up psychiatric evaluation, he expressed that it was maladaptive to obsess about his paranoid delusions. However, he complained that he felt restless, apathetic, and drowsy, in addition to gaining weight. Olanzapine was first reduced to 2.5 mg/day, and then replaced with aripiprazole 12 mg/day.

Three months later (November 2011), he confided that he had a history of 5-methoxy-*N*,*N*-diisopropyltryptamine (5-MeO-DIPT) ingestion, which he began abusing at 18 or 19 years old. He used this substance approximately 10 times and quit when he was 21 years old. However, about 1 year prior to his initial presentation, he had started ingesting a commercially available aromatic liquid for the purposes of recreation drug use, consuming it regularly (about 20 times) until one month ago (October 2011).

**Figure 1:**
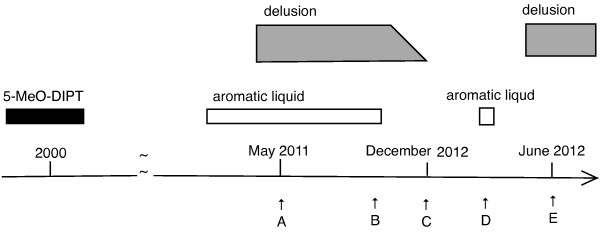
**Time course of drug ingestion and delusional state. A**. May 2011. First visit. **B**. October 2011. Quit aromatic liquid. **C**. December 2011. Last visit from first episode. **D**. March 2012. Restarted consuming aromatic liquid. **E**. June 2012. First visit from second episode.

The patient’s last visit from the initial psychotic episode was in December 2011. In January of the following year, the patient failed to attend his follow-up appointment and he was lost to follow-up. In June 2012, his mother informed us that his psychosis had relapsed, although he appeared to be normal in the intervening months. The patient presented to us again in July 2012, claiming that he was being harassed by people around him and that this was related to suspicious activity with his phone and computer. He also expressed that he was again suspicious of his wife. He confided that he had consumed another commercially available aromatic liquid twice in March. He was started on aripiprazole 6 mg/day, remaining in his delusional state through the end of September 2012 despite continuing on the medication. Figure 1 shows the time course of the patient’s drug ingestion and delusional state.

The brand names of the first aromatic liquids he consumed was “GT 5me0 (Citrus Paradisi FLAVOR)”, though their chemical ingredients were not clear. The patient had purchased both 5-MeO-DIPT and these aromatic liquids via Internet for the purpose of recreational oral use.

## Discussion

It is often difficult to gather information about a history of drug abuse as patients often attempt to hide this from evaluators. In this case, despite conducting careful interviews, it took several months to identify the patient’s drug abuse. An evaluation of the chronological sequence of the patient’s condition showed that these legal aromatic liquids, which can be purchased easily on the Internet, were very likely the trigger for the patient’s delusional episodes, although according to users’ comments available on the Internet, the use of these aromatic liquids was not associated with delusion. We suspect that these liquids may contain a psychoactive substance, inducing his delusions. However, in each episode, the patient’s delusional state was prolonged on the order of months; we found no prior reports of prolonged delusions triggered by abusing legal substances.

We speculate that the patient’s past history of 5-MeO-DIPT abuse played an important role. 5-MeO-DIPT is a commonly abused hallucinogenic drug, illegal in many countries, including Japan since 2005. There are many reports of acute psychosis related to 5-MeO-DIPT use [4-7], and its psychotomimetic effects have been considered to be due to enhancement of serotonergic neurotransmission and/or trace amine receptors [8]. The patient’s recurrent abuse of 5-MeO-DIPT might have caused sensitization (or reverse tolerance). Sensitization is the amplification of a response following repeated administrations of a stimulus. Relapse of amphetamine psychosis due to this mechanism is well documented [9,10]. Similar relapses have been reported with 5-MeO-DIPT, with one case report describing a patient experiencing a “bad trip” after hearing unexpected news [4]. It is possible that sensitization is the basis of relapse, although this is not explicitly discussed. We speculate that our patient’s response to ingesting the aromatic liquids was amplified, given that he had repeatedly abused 5-MeO-DIPT in the past, resulting in prolonged delusions.

## Conclusion

5-MeO-DIPT was previously a popular drug of abuse, and it is highly probable that many past 5-MeO-DIPT users remain sensitized. This is an important latent factor underlying subsequent episodes of drug-induced psychoses. Psychiatrists should consider the possibility of 5-MeO-DIPT sensitization when assessing patients with acute psychoses.

We hypothesize that sensitization caused by 5-MeO-DIPT exists not only in this case but also in many other cases of acute psychotic episodes in drug abusers. Provided that more cases are added and sensitization caused by 5-MeO-DIPT is clarified, further understanding may be available in the field of psychosis related to substance abuse.

### Informed consent

Written informed consent was obtained from the patient for publication of this case report and all accompanying images. A copy of the written consent is available for review by the Editor-in-Chief of this journal.

## Competing interests

None of the authors has any financial conflicts or competing interests to disclose.

## Authors’ contributions

YF conceived of the case report, performed the literature search, and drafted and revised the manuscript. TN performed the literature search and revised the manuscript. All authors read and approved the final draft.
